# PanExplorer: a web-based tool for exploratory analysis and visualization of bacterial pan-genomes

**DOI:** 10.1093/bioinformatics/btac504

**Published:** 2022-08-02

**Authors:** Alexis Dereeper, Marilyne Summo, Damien F Meyer

**Affiliations:** CIRAD, UMR ASTRE, F-97170 Petit-Bourg, France; French Institute of Bioinformatics (IFB)—South Green Bioinformatics Platform, Bioversity, CIRAD, INRAE, IRD, F-34398 Montpellier, France; French Institute of Bioinformatics (IFB)—South Green Bioinformatics Platform, Bioversity, CIRAD, INRAE, IRD, F-34398 Montpellier, France; CIRAD, UMR AGAP, F-34398 Montpellier, France; CIRAD, UMR ASTRE, F-97170 Petit-Bourg, France

## Abstract

**Motivation:**

As pan-genome approaches are largely employed for bacterial comparative genomics and evolution analyses, but still difficult to be carried out by non-bioinformatician biologists, there is a need for an innovative tool facilitating the exploration of bacterial pan-genomes.

**Results:**

PanExplorer is a web application providing various genomic analyses and reports, giving intuitive views that enable a better understanding of bacterial pan-genomes. As an example, we produced the pan-genome for 121 *Anaplasmataceae* strains (including 30 *Ehrlichia*, 15 *Anaplasma*, 68 *Wolbachia*).

**Availability and implementation:**

PanExplorer is written in Perl CGI and relies on several JavaScript libraries for visualization (hotmap.js, MauveViewer, CircosJS). It is freely available at http://panexplorer.southgreen.fr. The source code has been released in a GitHub repository https://github.com/SouthGreenPlatform/PanExplorer. A documentation section is available on PanExplorer website.

## 1 Introduction

In the past decade, the pan-genome concept has been largely employed to investigate the bacterial comparative genomics and evolution analyses (Hyun *et al.*, 2022; Jonkheer *et al.*, 2021; Vernikos, 2020). Many programs have been developed for this purpose such as Roary (Page *et al.*, 2015) or more recently PanACoTA (Perrin *et al.*, 2021), and a need is still present for the efficient storage, exploitation and visualization of data derived from pan-genome analyses. To address this question, we developed a web-based application, PanExplorer, which performs online pan-genome analysis and displays resulting information as a comprehensive and easy-to-use solution, through several modules facilitating the exploration of gene clusters and interpretation of data. Several web applications have been recently published for this purpose. PanX (Ding *et al.*, 2018) is a sophisticated web application for browsing among clusters after pre-computed pan-genome analyses, but it does not allow matrix visualization, synteny exploration nor genome import. On the other hand, PGAweb (Chen *et al.*, 2018), PGAPX (Zhao *et al.*, 2018) and PanWeb (Pantoja *et al.*, 2017) are web servers for running online the PGAP software (Zhao *et al.*, 2012) on users’ Genbank files, but output files and images cannot be manipulated and browsed interactively. Thus, in contrast to the existing web-based tools for exploring pan-genomes, PanExplorer brings together in a single web application a whole set of data representation modules that allow to view pan-genomic information from different angles and offers the possibility to submit online a customized selection of bacteria strains if completely assembled and annotated.

## 2 The PanExplorer application

**Fig. 1.**
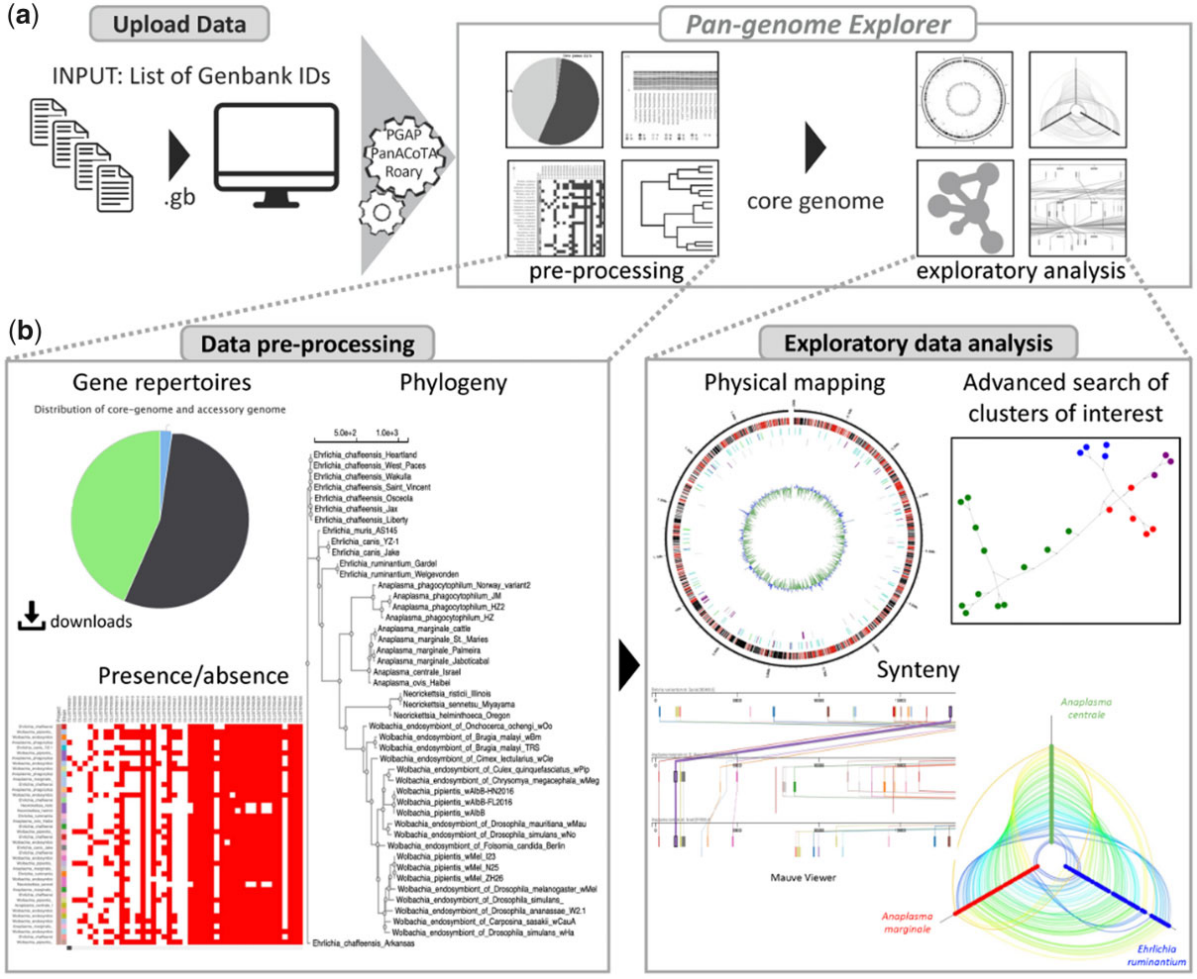
The PanExplorer application process. (**A**) Overview of the process: data upload, pre-processing using a PGAP/Roary/PanACoTA-based pipeline, data exploration. (**B**) After data processing, the application provides an overview of results, this includes PAV matrix, distribution of core and accessory genome and global phylogeny. Exploratory analysis consists of Circos representations of core-genes and strain-specific genes, synteny investigation or visual inspection of clusters of interest

The application takes a list of GenBank identifiers as input and the server will execute successively: (i) retrieval of the corresponding published complete genome sequences and their annotations, (ii) gene clustering and pan-genome analysis using PGAP (Zhao *et al.*, 2012), Roary (Page *et al.*, 2015) or PanACoTA (Perrin *et al.*, 2021), (iii) attribution of the Clusters of Orthologous Groups (COG) functional categories using RPSblast against the COG database (Tatusov *et al.*, 2000) and (iv) GC content calculation using SkewIT (Lu *et al.*, 2020) (Fig. 1A). The PanExplorer application is developed in Perl CGI, web interactivity is allowed by external JavaScript libraries: hotmap.js (https://github.com/nconrad/hotmap), MauveViewer (https://github.com/PATRIC3/mauve-viewer), CircosJS (https://github.com/nicgirault/circosJS), MSAViewer (Yachdav *et al.*, 2016), phylotree.js (Shank *et al.*, 2018) and D3 (Bostock *et al.*, 2011).

## 3 Interactive data exploration

### 3.1 Pan-genome visualization

As a presence/absence variation (PAV) matrix (Fig. 1B) using the hotmap.js javascript library. This overview allows to easily identify and distinguish core-genes (present in all strains), dispensable genes (genes from the accessory genome) and strain-specific genes. Furthermore, this section allows a particular cluster of interest to be selected for further investigation and displayed in detail (Fig. 1B). This section plots the distribution of COG function categories for each analyzed genome and highlights potentially over- or under-represented functional category in given strains. In addition, it also reports in a table list COG and COG categories assigned for each gene cluster of the core-genome.

### 3.2 Circos

The physical map of core-genes and strain-specific genes can be displayed as a circular genomic representation (Krzywinski *et al.*, 2009) (Fig. 1B), for each genome taken independently. Each gene is colorized according to its COG category. It also plots the GC skew values, in sliding windows along the genome.

### 3.3 Synteny

The conservation of gene order between genomes can be investigated using two graphical representations (Fig. 1B). A Hive Plot built with d3.js (Bostock *et al.*, 2011) is displayed for a global macro-synteny overview, allowing to detect rearrangements between strains (translocation, duplication or inversion) while a Mauve viewer (Darling *et al.*, 2004) allows the zoom in depth until giving access to the gene information. For both representations, each cluster of the core-genome is materialized as a link between genomes. Comparison is possible after the selection of three genomes among those available in the project.

### 3.4 Visual inspection of a specific cluster

PanExplorer offers the possibility to focus on any cluster of the pan-genome, and get access to its composition in terms of protein or DNA sequences, to their alignment using Muscle (Edgar *et al.*, 2004) and subsequent gene-based phylogeny (NJ distance tree) or colorized minimum spanning network (Fig. 1B). This examination can be reached from any cluster tables, outputted either from the cluster or gene search panel, or directly after clicking on a gene from Circos or pan-genome matrix overview.

### 3.5 Dynamic reevaluation using a subset of genomes

The application allows to reduce the PAV matrix after the selection of a subset of strains from the original collection of genomes (e.g. for focusing on one species or one particular phenotype), which finally results to a different list of genes defined as core-genes.

## 4 Conclusions

PanExplorer offers broad-spectrum of tools for easily exploring pan-genomes for scientists without programing skills (automatization of process, no installation is required). Thanks to a connection to NCBI ftp server, the application guarantees an up-to-date availability of public genomes, to be analyzed on-the-fly, and represents a versatile tool for genome exploration filling a need for bacteriologist community. Among perspectives and further development, new functionalities might be implemented shortly such as online pan-Genome Wide Association Studies (pan-GWAS) based on Scoary software (Brynildsrud *et al.*, 2016) or COG statistical enrichment studies. By allowing visualization of high dimensional data, PanExplorer can assist investigations of pan-genome dynamics of various bacterial lifestyles. It combines efficiently in-depth search of individual genes and their mutations as well as broad examination of pan-genomes. With routine publication of bacterial genomes, PanExplorer is now a web application of choice to assist microbiological research for a better control of bacterial infectious diseases.
